# Physicians payment in the United States between 2014 and 2018: An analysis of the CMS Open Payments database

**DOI:** 10.1371/journal.pone.0252656

**Published:** 2021-06-02

**Authors:** Raphael E. Cuomo, Mingxiang Cai, Neal Shah, Tim K. Mackey

**Affiliations:** 1 Global Health Policy and Data Institute, San Diego, CA, United States of America; 2 Department of Anesthesiology, San Diego School of Medicine, University of California, San Diego, CA, United States of America; 3 Department of Anthropology, Global Health Program, University of California, San Diego, CA, United States of America; 4 S-3 Research LLC, San Diego, CA, United States of America; 5 Department of Healthcare Research and Policy, San Diego—Extension, University of California, San Diego, CA, United States of America; 6 Division of Infectious Disease and Global Public Health, Department of Medicine, San Diego School of Medicine, University of California, San Diego, CA, United States of America; University of Utah, UNITED STATES

## Abstract

The Open Payments database reports payments made to physicians by industry. Given the potential for financial conflicts of interest relating to patient outcomes, further scrutiny of these data is valuable. Therefore, the objective of this study was to analyze physician-industry relationships by specialty type, payment type, geospatial trend, and longitudinal trend between 2014–2018. We conducted an observational, retrospective data analysis of payments from the Open Payments database for licensed United States physicians listed in the National Plan & Provider Enumeration System (NPPES). Datasets from 2013–2018 were joined using the Python programming language. Aggregation and sub-setting by characteristics of interest was done in R to calculate means and frequencies of reported general physician payments from industry across different specialties, locations, timeframes, and payment types. Normalization was applied for numbers of physicians or payments. Geospatial statistical hot spot analysis was conducted in ArcGIS. 51.73 million payment records were analyzed. In total, 50,047,930 payments were issued to 771,113 allopathic or osteopathic physicians, representing $8,702,631,264 transferred from industry to physicians over the five-year period between 2014 and 2018. The mean payment amount was $179, with a standard deviation of $12,685. Variability in physicians’ financial relationships with industry were apparent across specialties, regions, time, and payment type. A limited match rate between records in the NPPES and Open Payments databases may have resulted in selection bias of trends related to physician characteristics. Further research is necessary, particularly in the context of changing industry payment trends and public perceptions of the appropriateness of these relationships.

## Introduction

The influence that non-clinical factors may have on physician practices has long been debated by the medical community, policymakers, the media, and the general public [1]. Specifically, financial incentives and conflicts-of-interest (COI) have been implicated as important factors that can influence physician decision-making, whether in private practice, large hospital systems, or academic medicine settings [2, 3]. Specifically, transfers of value to physicians from pharmaceutical companies, biotechnology companies, device companies, and other medical manufacturers can play a role in influencing prescribing patterns, rationale selection, and patient outcomes leading to increased scrutiny, calls for regulation and transparency, and enforcement actions (e.g., including through prosecutions under the Anti-Kickback Statute and False Claims Act) [4, 5].

Financial relationships between physicians and various industry actors have historically been widespread, but also differ significantly in their characteristics of types of relationships, payments categories, and value/amount [6]. Studies have shown that many patients acknowledge physician-industry gifts and contributions as common in medicine; consequently, a majority of these patients demonstrate lower levels of trust for physicians and the healthcare system as a whole [7]. Such concerns led to the enactment of the Physician Payments Sunshine Act in 2010 as section 6002 of the Affordable Care Act [8]. This legislation mandates that biomedical manufacturers and other group purchasing organizations disclose all transfers of value to physicians or teaching hospitals, under penalty of law [9].

Examples of transfers of value include direct payments and indirect payments from a company to the physician, along with ownership stake. Reported payments are categorized by the nature of the value transferred. Various types are included, such as “current or prospective ownership or investment interest,” “food and beverage,” and “consulting fee.” The *de minimis* threshold for an individual transfer was $10.00 when the law went into force for the October 1, 2013 –December 31, 2013 data disclosure period, and has most recently risen to $10.97 for the January 1, 2020 –December 31, 2020 disclosure period [10].

These transfers of value are reported, adjudicated, and then disclosed on a public database called Open Payments, managed by the Center for Medicare & Medicaid Services (CMS) [11]. Research using this database can uncover patterns warranting investigation into categories of physicians (e.g., by specialty, type of payment, or geographic area) who receive payments and elucidate differences in physician-industry relationships across specialty areas. The identity of individual physicians is publicly available, as well as the address of their practice and their medical specialty. Previous studies that have been conducted using the Open Payments data have primarily focused on specialty-specific payments in specific reporting years [12–16], with few tracking longitudinal changes over a multiyear reporting period. Furthermore, few studies have assessed geospatial variability in payments to physicians, though one study found a positive association between opioid-related marketing and one-year time-lagged opioid prescribing and mortality at the county-level [17].

Building on this prior research, in this study we assessed variations in payments in the Open Payments database over the 5-year period from 2014–2018, inclusive. Our study is unique in that it includes comparisons across sub-specialties of all Allopathic & Osteopathic physicians, calendar quarters, geospatial boundaries, payment types, as well as numerous combinations of these variables. Specifically, in this study we assess variation across categories at the payment level, the recipient level, and the aggregate; and we assess geospatial variability through novel applications of geospatial statistics.

## Materials and methods

### CMS Open Payment data

Open Payment reports made by industry (including “applicable manufacturers” and “applicable group purchasing organizations”, also known as “Reporting Entities”) operating in the United States to covered recipients (i.e., any licensed physician in the United States, except for a physician who is a bona fide employee of the applicable manufacturer reporting a payment) from 2014–2018 were extracted from publicly available datasets on the CMS website for each year. In this study, our analysis is limited to the general payments category across a five-year period and we note that physicians who do not receive payments are not included in the CMS database. Therefore, data and payments related to ownership and investment interests were removed from the general payment file from CMS for the five-year period, and this file was merged into a master file for analysis.

### External data

To study the population of physicians who have received payments from Reporting Entities in the past five years, we integrated data from National Plan & Provider Enumeration System (NPPES) to match detailed profiles of physicians for further aggregation. Each record from the NPPES database represents a physician with a unique National Provider Identifier (NPI), which is different from the physicians’ unique identifier Physicians Profile ID (PPI) used in the Open Payment datasets. To enable profile referencing from the Open Payment physician records, we created a one-to-one mapping method from PPI to NPI by matching physicians’ name and address (**S1 Fig**). Specifically, the common attributes used to produce the mapping were first name, middle name, last name, practice address, and practice zip code. We matched by employing multiple sequential criteria, from strict to relaxed, to facilitate a higher matching rate while also facilitating matching quality. The strictest criterion was a full match on all of the attributes mentioned above. However, due to the possible inaccuracy in attributes such as address, fewer than 50% of Allopathic & Osteopathic physicians were matched. We then relaxed criteria so that zip code was used instead of full address and middle initial was used instead of full middle name. Once the mapping was created, we were able to aggregate payment data by the dimensions of physician profile (e.g., by specialties following NPPES provider taxonomy). In addition, payments made in years after 2014 were adjusted for inflation using annual rates obtained from the Federal Reserve Bank of St. Louis, thereby converting all dollar amounts to 2014 dollars.

### Analyses

Aggregation functions were written in R to assess variability across physician specialty, time, physician location, and payment type. Averages, sums, and counts were used to observe discrepancies across categories; normalization of averages was conducted at the transaction and recipient levels. Specifically, mean dollars per payment, mean dollars per physician, aggregate dollars, and count of payments were calculated to answer as set of five study questions:

1. **Payments by Specialty:** What specialty had the highest/lowest payment amount, average payment, average payment per physician, and number of payments? For the specialty with the highest average payment per physician, what was the state and payment category in which they were paid the most?
2. **Payments Over Time and Space:** What was the average quarterly change in payments over the five-year period? What state had the highest/lowest average quarterly growth/decline? What state had the highest/lowest payment amount, average payment, average payment per physician, and number of payments? What areas had statistically significant geospatial clustering, and how did geospatial clustering change over time?
3. **Payments by Type:** What payment type had the highest/lowest average payment, average payment per physician, aggregate payment amount, and number of payments?
4. **Payment Combinations:** What was the highest/lowest average payment, average payment per physician, aggregate payment amount, and number of payments for the combinations of specialty and payment type, state and payment type, and state and physician specialty?

Results were not reported when the highest or lowest value in a given category was based upon a sample size of under 30 payments. In addition, we assess the number of very high payments by reporting the number of individual payments two standard deviations above the mean. Pearson’s correlation coefficient was computed to determine the association between mean payment per physician and a mean years of practice over the five-year time frame. Furthermore, hot spot analysis was conducted for all available US zip codes by obtaining *z*-scores for the Getis-Ord Gi* statistic to enable visualization of a gradient from low-value clustering (i.e., “cold” spots) to high-value clustering (i.e., “hot” spots) among the aggregation of transactions across the five-year study period. Hot spot analyses were further stratified by year to relay variation in geospatial clustering over time. Statistical analyses were conducted in R v3.6.0 (R Foundation for Statistical Computing: Vienna, Austria), and geospatial visualization was done using ArcGIS (Esri: Redlands, CA).

## Results

Excluding payments of ownership and investment interests, there were 50,047,930 payments issued to 771,113 allopathic or osteopathic physicians, representing $8,702,631,264 transferred from industry to physicians over the five-year period between 2014 and 2018. The mean payment amount was $179, with a standard deviation of $12,685. Using a threshold of two standard deviations above the mean ($25,548), there were 19,549 (0.038%) payments of exceptionally high value. There was a very weak, although statistically significant, correlation between magnitude of individual payments transacted and corresponding physicians’ years of practice (ρ = 0.0014, *p*<0.05). However, the correlation between mean payment per physician and physician years of practice was not statistically significant (ρ = 0.0012, *p* = 0.35), potentially indicating that the possible small effect from physician experience on individual payment amount disappeared when aggregating over all types of payments. Furthermore, the mean amount received per physician was $11,285, with a standard deviation of $252,213; the mean dollars received by a state (including DC but excluding overseas territories) was $177,693,306, with a standard deviation of $228,171,439; and the mean dollars transferred per quarter was $462,049,096, with a standard deviation of $40,092,150.

### Payments by specialty

There were 539 provider types represented in the Open Payments database. This study assessed types within the top-level category of “Allopathic & Osteopathic Physicians” (thereby excluding other top-level categories such as Chiropractic Providers, Nursing Providers, and Suppliers). Allopathic and Osteopathic Physicians were further categorized into 36 top-level specialties and, where applicable, 225 more granular specialty levels (**S1 Table**). Among all 36 top-level specialties categorized within the “Allopathic & Osteopathic Physician” category, Orthopedic Surgery received the highest amount per physician, though did not have an especially large number of physicians represented ($\bar{x}$ = $52,388; n = 40,014 physicians; **Table 1**).

**Table 1:** Variability in mean payment per physician, number of physicians, and aggregated payments for transactions in the Open Payments database, 2014–2018, for each top-category specialty available for allopathic and osteopathic physicians.

Physician Specialty	Amount per Physician	Number of Physicians	Payments per Physician	Total Amount Paid
Orthopaedic Surgery	$52,388	40,014	39.01	$2,096,265,662
Clinical Pharmacology	$36,931	93	20.74	$3,434,599
Neurological Surgery	$31,881	13,666	30.85	$435,684,863
Thoracic Surgery (Cardiothoracic Vascular Surgery)	$29,618	5,347	63.37	$158,366,767
Plastic Surgery	$15,020	7,229	29.44	$108,579,387
Colon & Rectal Surgery	$13,516	1,935	45.38	$26,152,609
Nuclear Medicine	$12,924	1,485	20.49	$19,191,843
Medical Genetics	$12,315	690	20.90	$8,497,630
Urology	$11,988	12,990	83.27	$155,720,221
Neuromusculoskeletal Medicine, Sports Medicine	$11,823	410	33.51	$4,847,593
Ophthalmology	$11,445	26,069	31.10	$298,358,424
Psychiatry & Neurology	$10,738	67,867	67.26	$728,738,497
Dermatology	$10,673	22,277	72.50	$237,771,826
Allergy & Immunology	$9,108	7,052	63.60	$64,226,239
Radiology	$8,802	33,747	15.45	$297,055,894
Internal Medicine	$8,703	338,043	66.94	$2,942,106,974
Surgery	$8,022	47,039	29.05	$377,342,357
Transplant Surgery	$7,838	952	18.21	$7,461,827
Pain Medicine	$5,583	8,747	48.23	$48,833,062
Pathology	$4,858	12,491	7.08	$60,685,903
Independent Medical Examiner	$4,479	110	6.86	$492,656
Obstetrics & Gynecology	$3,863	52,823	30.73	$204,066,159
Physical Medicine & Rehabilitation	$3,773	12,590	38.81	$47,498,413
Otolaryngology	$3,771	13,759	24.61	$51,879,410
Neuromusculoskeletal Medicine & OMM	$3,641	1,053	29.74	$3,833,535
Phlebology	$3,411	158	12.17	$538,969
Oral & Maxillofacial Surgery	$3,314	1,372	13.04	$4,546,451
Preventive Medicine	$3,126	3,299	10.18	$10,312,485
Anesthesiology	$3,027	45,348	19.54	$137,277,135
Pediatrics	$2,690	69,754	17.67	$187,628,302
Family Medicine	$2,052	164,912	59.50	$338,336,756
Emergency Medicine	$1,990	35,230	7.49	$70,109,900
General Practice	$1,462	66,421	9.39	$97,114,609
Electrodiagnostic Medicine	$1,303	31	13.16	$40,404
Hospitalist	$644	12,191	10.07	$7,851,913
Legal Medicine	$491	270	5.01	$132,656

A red-yellow-green gradient was applied, per column, to illustrate low-medium-high relative amounts.

At the most granular specialty level, the specialty with the highest paid average physician was Adult Reconstructive Orthopedic Surgery ($\bar{x}$ = $146,032; n = 1,338 physicians), although Pediatric Critical Care received the highest average individual payment amount ($\bar{x}$ = $5,870; n = 5,219 payments). Pediatric Medical Toxicologists received the lowest average payment amount per payment ($\bar{x}$ = $22; n = 40 payments). The specialty with the highest amount of individual payments was Family Medicine, with 9,589,197 payments. Overall, the specialty with the highest aggregate payment amount was Orthopedic Surgery (non-specialty; $1,261,714,298).

The state where Adult Reconstructive Orthopedic Surgeons received the highest payments was Arkansas ($\bar{x}$ = $9,529, n = 736 payments). The highest category of payment type for Adult Reconstructive Orthopedic Surgeons was “royalty or license” ($\bar{x}$ = $46,888, n = 3,120 payments).

### Payments over time and space

Overall, the total amount of payments transferred exhibited an increasing trend over the twenty-quarter period analyzed, growing an average of $5,068,804 (2.06%) per quarter. However, growth in payments exhibited wide variation across states. The state with the highest average quarterly growth was Montana (36.15% growth per quarter), and the state with the lowest was Ohio (0.32% growth per quarter).

**Fig 1:**
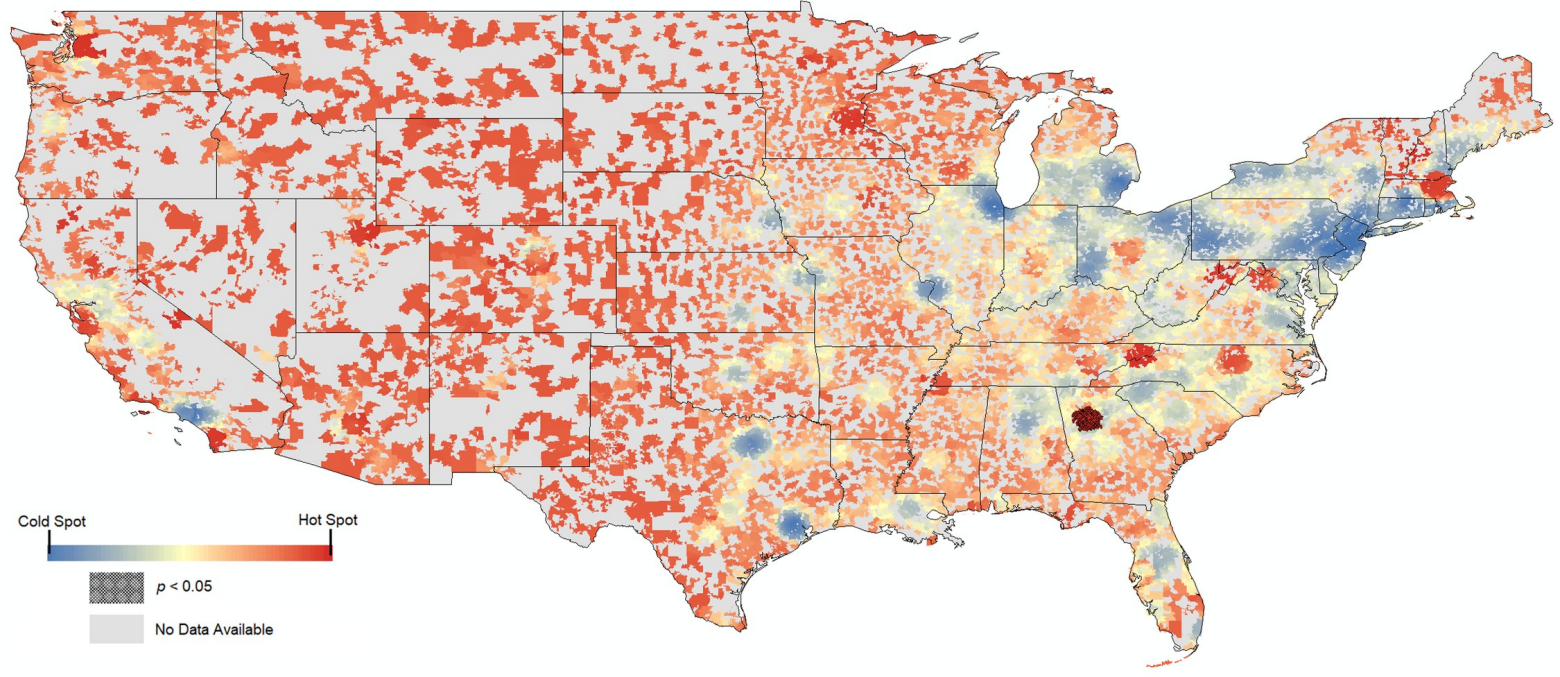
In blue-red gradient, results of a hot spot analysis using zip code averages for payment amount per physician in the contiguous United States. Hatched overlay represents zip codes where hot spot reached statistical significance, and gray zip codes are those with no data. *Cartographic boundaries obtained from the U*.*S*. *Census Bureau and reprinted with permission in accordance with OMB Memorandum M-10-06 and Executive Order 13642*.

The state having the highest-paid average physician was Tennessee ($\bar{x}$ = $17,596; n = 17,912 physicians), though Vermont had the highest individual average payment amount ($\bar{x}$ = $787; n = 9,113 payments). Conversely, the state with the lowest-paid average physician was Alaska ($\bar{x}$ = $2,052; n = 1,739 physicians), and the state with the lowest average payment was Arkansas ($\bar{x}$ = $68; n = 500,908 payments). The state receiving the greatest total amount of payments was California ($1,200,395,000), which also had the highest number of individual payments (5,154,789). The state receiving the lowest aggregate payment was Alaska ($3,568,673), with the state receiving the lowest number of payments being Vermont (9,113). Hot spot analysis of individual payment amounts over the five-year period at the zip code level revealed a high degree of heterogeneity, with a statistically significant hot spot cluster observed among zip codes in the Atlanta, Georgia metropolitan area (**Fig 1**).

**Fig 2:**
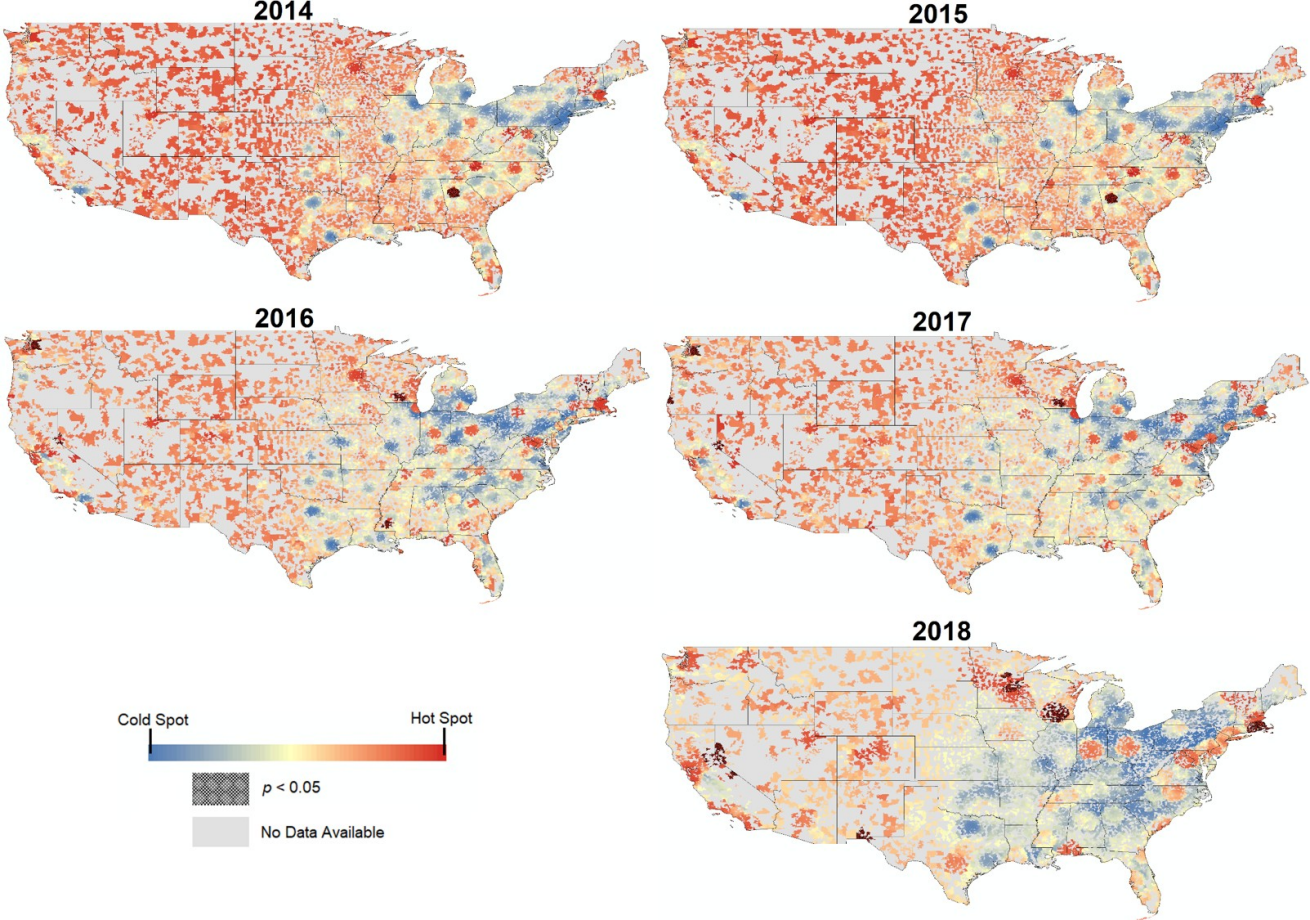
In blue-red gradient, annual variation in geospatial clustering of average individual payments per zip code, with hatched overlay representing statistical significance. Cartographic boundaries obtained from the U.S. Census Bureau and reprinted with permission in accordance with OMB Memorandum M-10-06 and Executive Order 13642.

Annual stratification of geospatial clustering revealed a number of trends over time (**Fig 2**). Over the five-year period, the eastern half of the contiguous United States appeared to increasingly exhibit lower amounts of industry payments when compared to the western half of the country. The hot spot appearing for zip codes in the Atlanta metropolitan area was evident in 2014 and 2015 but disappeared thereafter. Conversely, no statistically significant hot spot was detected on the western half of the contiguous United States in 2014 and 2015, but a hotspot was observed in the Seattle, Washington area in 2016 and 2017; in the Portland, Oregon area in 2017; on part of the New Mexico-Texas border in 2018; and in the Reno, Nevada area between 2016–2018. However, a statistically significant hotspot was observed in the Milwaukee, Wisconsin area between 2016–2018, and a hotspot emerged in the Boston, Massachusetts area in 2018. Detection of these geospatial hotspots warrants further analysis of specific influx of types of payments to physician recipients that may have generated these patterns over time.

### Payments by type

When comparing across payment types, the category of “food and beverage” had the highest total number of payment transfers (45,786,759 payments; **Table 2**). However, “royalty or license” exhibited the highest average payment amount ($42,714; n = 54,548 payments), with “food and beverage” exhibiting the lowest average payment amount ($23; 45,786,759 payments). Similarly, the “royalty or license” category had the highest average amount per recipient ($758,942; n = 3,070 physicians), with “entertainment” having the least ($153; n = 8,260 physicians). The category with the highest total amount of value transferred was “compensation for services other than consulting, including serving as faculty or as a speaker at a venue other than a continuing education program” ($2,627,532,000), with “food and beverage” being the category with the highest total number of payments (45,786,759).

**Table 2:** For each type of payment category among general payments in the Open Payments database, 2013–2018, the mean payment per physician, the number of physicians receiving payments, and the total amount of money transacted.

Nature of Payment	Amount per Physician	Number of Physicians	Total Amount Paid
Royalty or License	$722,898	3,417	$2,470,142,824
Compensation for services other than consulting, including serving as faculty or as a speaker at a venue other than a continuing education program	$36,754	76,069	$2,795,846,598
Consulting Fee	$20,769	91,800	$1,906,582,821
Charitable Contribution	$12,948	1,436	$18,592,905
Compensation for serving as faculty or as a speaker for an accredited or certified continuing education program	$12,946	2,850	$36,894,992
Grant	$10,640	9,176	$97,633,573
Compensation for serving as faculty or as a speaker for a non-accredited and noncertified continuing education program	$10,441	11,045	$115,316,172
Honoraria	$9,068	28,146	$255,234,328
Travel and Lodging	$5,265	180,736	$951,610,060
Food and Beverage	$1,224	972,186	$1,190,126,558
Gift	$788	72,722	$57,309,996
Education	$627	343,477	$215,344,106
Entertainment	$155	23,152	$3,581,211

A red-yellow-green gradient was applied, per column, to illustrate low-medium-high relative amounts.

### Payment combinations

Arizona Pediatric Critical Care physicians were the specialty-state combination with the highest mean payment ($\bar{x}$ = $280,204; n = 102 payments), with also the highest mean amount per recipient ($\bar{x}$ = $952,693; n = 30 physicians). The specialty-state combination with the highest total amount of value transferred was for payments to Orthopedic Surgeons in Texas ($178,768,903), although the specialty-state combination with the highest number of payments was for Internal Medicine physicians in California (887,865).

The combination of specialty and payment type with the lowest mean payment was “gift” for Clinical & Dermatological Immunology ($\bar{x}$ = $1.83; n = 48 payments). The combination with the highest number of total payments was “food and beverage” payments to Family Medicine physicians (9,234,821). “Royalty or license” payments to Orthopedic Surgeons exhibited the greatest aggregation of money transferred among all combinations of specialty and payment type, with a total of $914,501,294.

The combination of state and payment type with the highest average payment was “royalty or license” to recipients in Tennessee ($\bar{x}$ = $127,171; n = 1,406 payments), which also had the highest average payment per physician ($\bar{x}$ = $2,322,108; n = 77 physicians). Contrarily, “food and beverage” payments to recipients in Arkansas had the lowest amount per payment ($\bar{x}$ = $17; n = 474,501 payments). Payments for “food and beverage” in California exhibited the highest number of payments across all state-type combinations (4,478,499), though the combination with the highest aggregate amount transferred was “compensation for services other than consulting, including serving as faculty or as a speaker at a venue other than a continuing education program” in California ($368,494,474).

## Discussion

Multiple studies have been conducted analyzing specialty-specific payment patterns between industry and physicians using the Open Payments database including but not limited to studies on orthopedic surgeons [12], emergency physicians [13], pediatricians [14], neurosurgeons [15], otolaryngologists [18], cardiologists [19], and ophthalmologists [20]. Additional research has also been conducted to further examine potential physician bias and conflicts of interest within particular specialty areas [21–23]. A recent systematic review also found that a large majority of studies found a positive association between industry payments and increased prescribing, including temporal and dose-response relationships [24]. Our study augments these results and found appreciable variation in physician payments observed across specialty, space, time, and type. Similar to prior studies, our findings also indicate that in order to fully understand macro trends in physician-industry relationships, careful contextualization of the types of payments received, the amounts and values that can potentially trigger COIs, and more detailed analysis of significant longitudinal or geographic shifts in spending is needed [24].

The highest aggregate payments from industry to physicians we observed in this study was for the specialties of Orthopedic Surgery. Traditionally, orthopedic surgeries and specialties within cardiovascular diseases—such as general cardiologists, interventional cardiologists, cardiothoracic surgeons, and cardiac electrophysiologists—conduct numerous procedures utilizing various medical devices and equipment, which may lead to a comparatively higher number of aggregate payments arising from nuances related to relationships with manufacturers and contracts for products used by physicians in these areas [25]. From a system-level perspective, Orthopedic Surgeons received a total of approximately $1.3 billion in transferred value, with relatively high individual payments and number of payments, with “Royalty or license” payments accounting for most of this amount. For the general Orthopedic Surgeon population, high payments were especially common in Texas.

Payments to Pediatric Critical Care physicians warrant additional examination, especially in Arizona where individual payments were high. Given that Pediatric Critical Care physicians in Arizona were our highest recorded specialty-state mean payment combination, additional research examining the possible primary drivers of this finding could provide additional contextualization of why this population of physicians seems to have inordinately high exposure to industry promotion activities. Previous open payment studies have found that Developmental and Endocrinologist Pediatricians received the highest percentage and highest median payment respectively, with most payments associated with attention-deficit hyperactivity disorders and vaccinations [14]. Further examination of differences between pediatric specialties and types of pharmaceutical and vaccines products subject to payment may help to further elucidate this high level of industry spend potentially impacting pediatric populations.

Though Family Medicine practitioners received a very high number of payments, these are for relatively low dollar amounts and are commonly for “food and beverage” items. Relatedly, a previous study found an association between industry-sponsored meals and increased rate of prescribing the specific brand-name pharmaceutical that was promoted, with higher frequency and higher dollar value of meals corresponding to higher prescription rates [26]. However, the actual impact of these small value payments as inducements is questionable, particularly in the context of other higher value spend we observed in other specialties that could result in more significant COIs or individual financial benefit.

Other notable regional variances and trends were also discovered in this analysis. Payments to Tennessee physicians were high, and Montana exhibited a rapid growth in total amount received over time. Further investigation should be conducted to determine the fundamental drivers of growth specific to these regions, though growth may be the result of development or relocation of new pharmaceutical and device companies or historic lower levels of promotion in these regions.

Our study also observed a relative shift in mean zip code-level transactions from the eastern half of the contiguous United States to the western half. However, the change in relative mean payment was not evenly distributed in either half of the country, with statistically significant hot spots forming in the eastern US later in the study period. Currently, no literature has thoroughly assessed why the breadth of general payments are notably different across US regions and states. Variability in industry payments to physicians across regions has implications for national and subnational policymaking, as policy measures can be taken to mitigate potential geospatial disparities in patient outcomes that could result from the influence of non-clinical factors on patient care decisions. More specifically, states may decide to respond with their own state-specific transparency and payment disclosure policies, similar to those in place prior to enactment of the Federal Sunshine Act and Open Payments system.

Payments in royalties each represented very large individual payments to physicians and constitute a fundamentally different form of COI compared to general payments in other categories, such as those consisting of food and beverages and entertainment. Specifically, royalty payments may be more directly tied to the outcome of prescribing rates or sales performance of a medical product being promoted, creating a situation where healthcare decisions are more intrinsically tied to individual financial benefit [27]. More detailed analysis of these types of relationships should be incorporated into future disclosure requirements, thereby allowing careful attention to reciprocity and actual prescribing behavior.

Additionally, we observed high amounts of value transferred for the category “compensation for services other than consulting”, which is inclusive of serving as a faculty of a medical congress or for speaking engagements that are not for continuing education. The details of this category seems unnecessarily opaque for the amount of spend observed, potentially necessitating more detailed information on the specific types of financial relationships and payments occurring in this category. Though payments for speaking engagements were not individually large, they represented the highest total amount of aggregate value transferred, warranting further examination into the purpose and intent of these engagements (i.e., for purposes of education or primarily as a vehicle to transfer value).

This study focused on assessing variations in industry payments to physicians according to physician specialty, time of receipt, physician location, and nature of payment. Further studies could also take into account the characteristics of payers to determine which manufacturers and drug and medical device product types appear to exert relatively high levels promotional spend and potential influence on providers, and whether there exists clustering of physician specialties with these manufacturer/product characteristics. Additionally, while this study assessed discrepancies between categories by aggregate dollars, mean dollars per payment, mean dollars per physician, and number of payments, further studies may seek to assess variation across specialties using additional metrics that take into account the number of recipients in a specialty as a fraction of the full number of practicing providers within a specialty.

### Limitations

This study has certain limitations. The two main databases consulted, the NPPES and Open Payments datasets, may have specific inaccuracies leading to potential errors regarding physician identification and incorrectly reported payments. Nevertheless, because Open Payments is still the most expansive and comprehensive database known for quantifying industry payments to physicians, it remains the primary data repository to conduct such research. Additionally, our data join methodology was not able to match all NPPES records with those in the Open Payments Database (match rate of 79% was achieved with missing records due to discrepancies between physician attributes between databases), which might have resulted in the influence of selection bias for trends associated with physician characteristics such as specialty or location.

## Conclusion

Despite the passage of the Sunshine Act, physician-industry financial relationships remain common in the United States healthcare system and are often cited as ways to spur innovation and provide practicing physicians with greater education, understanding, and familiarity of pharmaceuticals and devices [9]. However, concerns remain regarding potential COIs generated from these interactions and the effect it can have on healthcare spending, prescribing patterns and overutilization, the physician-patient relationship, and the overall delivery and quality of care [9]. Hence, the CMS Open Payments database is vital to ensuring appropriate transparency in order to better understand these relationships, particularly as they continue to evolve due to the “light they shine” on these relationships and subsequent public scrutiny. The framework of the Sunshine Act is also expanding to other areas of healthcare practice, including the addition of new covered recipient provider types (physician assistants, nurse practitioners, clinical nurse specialists, certified registered nurse anesthetists and anesthesiologist assistants, and certified nurse-midwives) starting in 2021 and has also been proposed to extend to other health policy areas (such as disclosure of direct-to-consumer advertising expenditures) [28, 29]. Future studies should continue to identify, characterize, and quantify the impact that “sunshine” has on industry, providers and patients. Consistent with the legislative intent of the Sunshine Act, the influence of these payments needs to be carefully monitored in order to ensure an optimal balance between physician and industry interactions, while also ensuring that these relationships take into consideration the best interests of the patient.

## Supporting information

S1 Fig Flow chart denoting step-by-step match rate between data for Allopathic & Osteopathic physicians in the Open Payments database and NPPES, with criteria denoted adjacent to arrows between running totals. (TIF)

S1 Table For general payments over the five-year period from 2014–2018, complete listing for number of physicians for all top-level categories and sub-categories for Allopathic and Osteopathic physicians in the Open Payments database. (DOCX)
