# Supplementary figures and images for: Physicians payment in the United States between 2014 and 2018: An analysis of the CMS Open Payments database
Source: PLoS One. 2021 Jun 2;16(6):e0252656. doi: 10.1371/journal.pone.0252656 (PMC8171935; doi:10.1371/journal.pone.0252656)

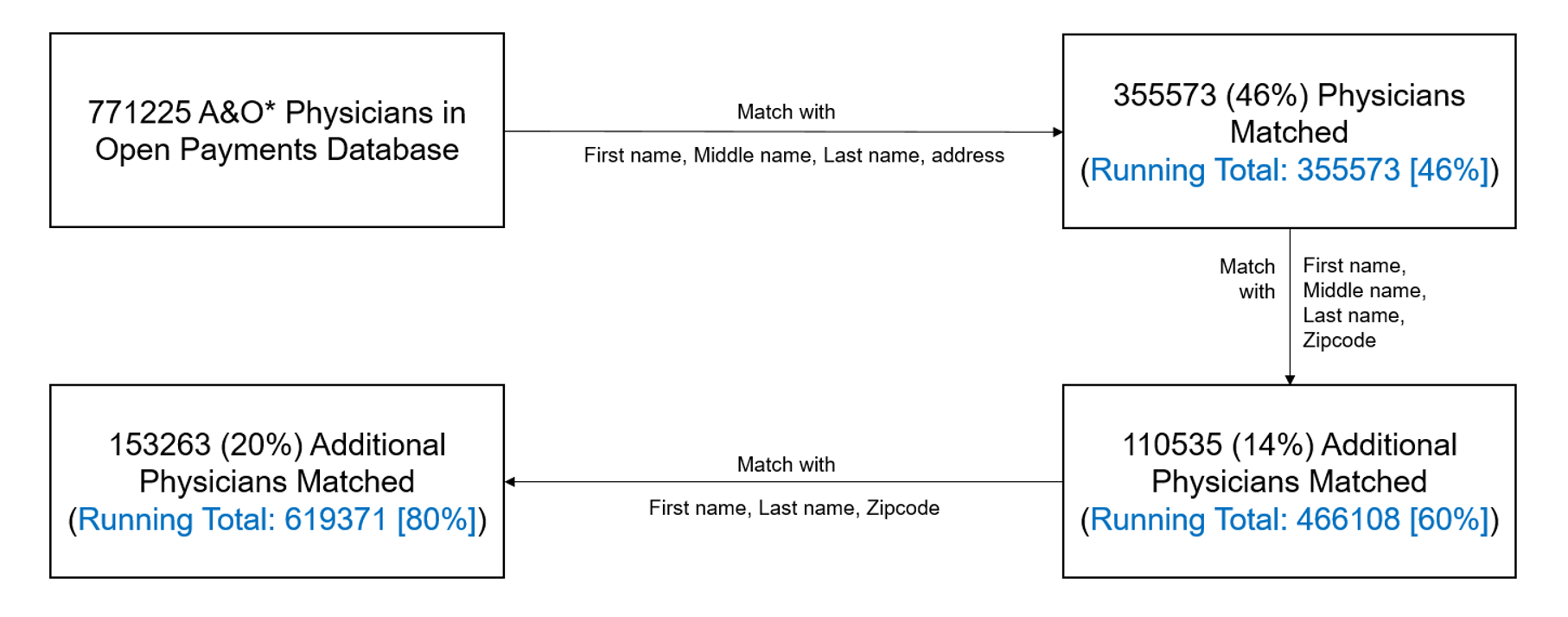

Supplement: S1 Fig — (TIF) [file pone.0252656.s001.tif]
