# Supplementary material for: Physicians payment in the United States between 2014 and 2018: An analysis of the CMS Open Payments database
Source: PLoS One. 2021 Jun 2;16(6):e0252656. doi: 10.1371/journal.pone.0252656 (PMC8171935; doi:10.1371/journal.pone.0252656)
Supplement: S1 Table — (DOCX) [file pone.0252656.s002.docx]

**S1 Table.** For general payments over the five-year period from 2014-2018, complete listing for number of physicians for all top-level categories and sub-categories for Allopathic and Osteopathic physicians in the Open Payments database

| **Top Category** | **Sub-Category** | **Number of Physicians** |
| --- | --- | --- |
| Allergy & Immunology | *No Specialty* | 4583 |
|  | Allergy | 2335 |
|  | Clinical & Laboratory Immunology | 134 |
| Anesthesiology | *No Specialty* | 37191 |
|  | Addiction Medicine | 110 |
|  | Critical Care Medicine | 960 |
|  | Hospice and Palliative Medicine | 57 |
|  | Pain Medicine | 6332 |
|  | Pediatric Anesthesiology | 698 |
| Clinical Pharmacology | *No Specialty* | 93 |
| Colon & Rectal Surgery | *No Specialty* | 1935 |
| Dermatology | *No Specialty* | 18789 |
|  | Clinical & Laboratory Dermatological Immunology | 241 |
|  | Dermatopathology | 734 |
|  | MOHS-Micrographic Surgery | 1102 |
|  | Pediatric Dermatology | 249 |
|  | Procedural Dermatology | 1162 |
| Electrodiagnostic Medicine | *No Specialty* | 31 |
| Emergency Medicine | *No Specialty* | 31545 |
|  | Emergency Medical Services | 2564 |
|  | Hospice and Palliative Medicine | 60 |
|  | Medical Toxicology | 87 |
|  | Pediatric Emergency Medicine | 448 |
|  | Sports Medicine | 342 |
|  | Undersea and Hyperbaric Medicine | 184 |
| Family Medicine | *No Specialty* | 156260 |
|  | Addiction Medicine | 592 |
|  | Adolescent Medicine | 705 |
|  | Adult Medicine | 1367 |
|  | Geriatric Medicine | 2247 |
|  | Hospice and Palliative Medicine | 378 |
|  | Obesity Medicine | 84 |
|  | Sleep Medicine | 323 |
|  | Sports Medicine | 2956 |
| General Practice | *No Specialty* | 66421 |
| Hospitalist | *No Specialty* | 12191 |
| Independent Medical Examiner | *No Specialty* | 110 |
| Internal Medicine | *No Specialty* | 175529 |
|  | Addiction Medicine | 361 |
|  | Adolescent Medicine | 442 |
|  | Adult Congenital Heart Disease | 9 |
|  | Advanced Heart Failure and Transplant Cardiology | 428 |
|  | Allergy & Immunology | 2771 |
|  | Cardiovascular Disease | 32012 |
|  | Clinical & Laboratory Immunology | 35 |
|  | Clinical Cardiac Electrophysiology | 3533 |
|  | Critical Care Medicine | 6362 |
|  | Endocrinology, Diabetes & Metabolism | 9269 |
|  | Gastroenterology | 20743 |
|  | Geriatric Medicine | 4424 |
|  | Hematology | 2648 |
|  | Hematology & Oncology | 15246 |
|  | Hepatology | 576 |
|  | Hospice and Palliative Medicine | 728 |
|  | Hypertension Specialist | 50 |
|  | Infectious Disease | 7897 |
|  | Interventional Cardiology | 12323 |
|  | Magnetic Resonance Imaging (MRI) | 102 |
|  | Medical Oncology | 7836 |
|  | Nephrology | 11185 |
|  | Obesity Medicine | 117 |
|  | Pulmonary Disease | 14703 |
|  | Rheumatology | 6721 |
|  | Sleep Medicine | 1516 |
|  | Sports Medicine | 312 |
|  | Transplant Hepatology | 165 |
| Legal Medicine | *No Specialty* | 270 |
| Medical Genetics | Clinical Biochemical Genetics | 57 |
|  | Clinical Cytogenetic | 21 |
|  | Clinical Genetics (M.D.) | 530 |
|  | Clinical Molecular Genetics | 22 |
|  | Molecular Genetic Pathology | 28 |
|  | Ph.D. Medical Genetics | 32 |
| Neurological Surgery | *No Specialty* | 13666 |
| Neuromusculoskeletal Medicine & OMM | *No Specialty* | 1053 |
|  | *No Specialty* | 410 |
| Nuclear Medicine | *No Specialty* | 769 |
|  | In Vivo & In Vitro Nuclear Medicine | 22 |
|  | Nuclear Cardiology | 544 |
|  | Nuclear Imaging & Therapy | 150 |
| Obstetrics & Gynecology | *No Specialty* | 39852 |
|  | Critical Care Medicine | 46 |
|  | Female Pelvic Medicine and Reconstructive Surgery | 627 |
|  | Gynecologic Oncology | 1717 |
|  | Gynecology | 5844 |
|  | Hospice and Palliative Medicine | 78 |
|  | Maternal & Fetal Medicine | 1530 |
|  | Obesity Medicine | 15 |
|  | Obstetrics | 1651 |
|  | Reproductive Endocrinology | 1463 |
| Ophthalmology | *No Specialty* | 24729 |
|  | Cornea and External Diseases Specialist | 56 |
|  | Glaucoma Specialist | 188 |
|  | Neuro-ophthalmology | 23 |
|  | Ophthalmic Plastic and Reconstructive Surgery | 221 |
|  | Pediatric Ophthalmology and Strabismus Specialist | 56 |
|  | Retina Specialist | 766 |
|  | Uveitis and Ocular Inflammatory Disease | 30 |
| Oral & Maxillofacial Surgery | *No Specialty* | 1372 |
| Orthopaedic Surgery | *No Specialty* | 27478 |
|  | Adult Reconstructive Orthopaedic Surgery | 1338 |
|  | Foot and Ankle Surgery | 1163 |
|  | Hand Surgery | 2548 |
|  | Orthopaedic Surgery of the Spine | 2355 |
|  | Orthopaedic Trauma | 882 |
|  | Pediatric Orthopaedic Surgery | 674 |
|  | Sports Medicine | 3576 |
| Otolaryngology | *No Specialty* | 10102 |
|  | Facial Plastic Surgery | 655 |
|  | Otolaryngic Allergy | 188 |
|  | Otolaryngology/Facial Plastic Surgery | 824 |
|  | Otology & Neurotology | 385 |
|  | Pediatric Otolaryngology | 536 |
|  | Plastic Surgery within the Head & Neck | 779 |
|  | Sleep Medicine | 290 |
| Pain Medicine | Interventional Pain Medicine | 2554 |
|  | Pain Medicine | 6193 |
| Pathology | Anatomic Pathology | 1254 |
|  | Anatomic Pathology & Clinical Pathology | 7360 |
|  | Blood Banking & Transfusion Medicine | 428 |
|  | Chemical Pathology | 23 |
|  | Clinical Informatics | 5 |
|  | Clinical Pathology | 809 |
|  | Clinical Pathology/Laboratory Medicine | 722 |
|  | Cytopathology | 503 |
|  | Dermatopathology | 380 |
|  | Forensic Pathology | 109 |
|  | Hematology | 467 |
|  | Immunopathology | 53 |
|  | Medical Microbiology | 51 |
|  | Molecular Genetic Pathology | 133 |
|  | Neuropathology | 121 |
|  | Pediatric Pathology | 73 |
| Pediatrics | *No Specialty* | 50763 |
|  | Adolescent Medicine | 2302 |
|  | Child Abuse Pediatrics | 16 |
|  | Clinical & Laboratory Immunology | 15 |
|  | Developmental Behavioral Pediatrics | 399 |
|  | Hospice and Palliative Medicine | 65 |
|  | Medical Toxicology | 6 |
|  | Neonatal-Perinatal Medicine | 3308 |
|  | Neurodevelopmental Disabilities | 292 |
|  | Pediatric Allergy/Immunology | 574 |
|  | Pediatric Cardiology | 1875 |
|  | Pediatric Critical Care Medicine | 1263 |
|  | Pediatric Emergency Medicine | 649 |
|  | Pediatric Endocrinology | 1636 |
|  | Pediatric Gastroenterology | 1527 |
|  | Pediatric Hematology-Oncology | 2017 |
|  | Pediatric Infectious Diseases | 753 |
|  | Pediatric Nephrology | 589 |
|  | Pediatric Pulmonology | 1144 |
|  | Pediatric Rheumatology | 325 |
|  | Pediatric Transplant Hepatology | 17 |
|  | Sleep Medicine | 79 |
|  | Sports Medicine | 140 |
| Phlebology | *No Specialty* | 158 |
| Physical Medicine & Rehabilitation | *No Specialty* | 8657 |
|  | Brain Injury Medicine | 10 |
|  | Hospice and Palliative Medicine | 55 |
|  | Neuromuscular Medicine | 115 |
|  | Pain Medicine | 2811 |
|  | Pediatric Rehabilitation Medicine | 173 |
|  | Spinal Cord Injury Medicine | 178 |
|  | Sports Medicine | 591 |
| Plastic Surgery | *No Specialty* | 6353 |
|  | Plastic Surgery Within the Head and Neck | 425 |
|  | Surgery of the Hand | 451 |
| Preventive Medicine | Aerospace Medicine | 262 |
|  | Clinical Informatics | 3 |
|  | Medical Toxicology | 18 |
|  | Obesity Medicine | 7 |
|  | Occupational Medicine | 1112 |
|  | Preventive Medicine/Occupational Environmental Medicine | 464 |
|  | Public Health & General Preventive Medicine | 1169 |
|  | Sports Medicine | 32 |
|  | Undersea and Hyperbaric Medicine | 232 |
| Psychiatry & Neurology | Addiction Medicine | 1988 |
|  | Addiction Psychiatry | 668 |
|  | Behavioral Neurology & Neuropsychiatry | 126 |
|  | Brain Injury Medicine | 2 |
|  | Child & Adolescent Psychiatry | 5684 |
|  | Clinical Neurophysiology | 1411 |
|  | Diagnostic Neuroimaging | 38 |
|  | Forensic Psychiatry | 489 |
|  | Geriatric Psychiatry | 859 |
|  | Hospice and Palliative Medicine | 25 |
|  | Neurocritical Care | 89 |
|  | Neurodevelopmental Disabilities | 29 |
|  | Neurology | 17973 |
|  | Neurology with Special Qualifications in Child Neurology | 1742 |
|  | Neuromuscular Medicine | 204 |
|  | Obesity Medicine | 25 |
|  | Pain Medicine | 298 |
|  | Psychiatry | 34905 |
|  | Psychosomatic Medicine | 237 |
|  | Sleep Medicine | 525 |
|  | Sports Medicine | 14 |
|  | Vascular Neurology | 536 |
| Radiology | Body Imaging | 967 |
|  | Diagnostic Neuroimaging | 89 |
|  | Diagnostic Radiology | 21649 |
|  | Diagnostic Ultrasound | 135 |
|  | Hospice and Palliative Medicine | 8 |
|  | Neuroradiology | 1082 |
|  | Nuclear Radiology | 345 |
|  | Pediatric Radiology | 395 |
|  | Radiation Oncology | 5034 |
|  | Radiological Physics | 17 |
|  | Therapeutic Radiology | 235 |
|  | Vascular & Interventional Radiology | 3791 |
| Surgery | *No Specialty* | 34223 |
|  | Hospice and Palliative Medicine | 75 |
|  | Pediatric Surgery | 906 |
|  | Plastic and Reconstructive Surgery | 2449 |
|  | Surgery of the Hand | 646 |
|  | Surgical Critical Care | 1406 |
|  | Surgical Oncology | 1431 |
|  | Trauma Surgery | 1045 |
|  | Vascular Surgery | 4858 |
| Thoracic Surgery (Cardiothoracic Vascular Surgery) |  | 5347 |
| Transplant Surgery | *No Specialty* | 952 |
| Urology | *No Specialty* | 12451 |
|  | Female Pelvic Medicine and Reconstructive Surgery | 140 |
|  | Pediatric Urology | 399 |
